# Walled-Off Pancreatic Necrosis Mimicking a Pancreatic Pseudocyst: A Diagnostic Pitfall With Therapeutic Implications

**DOI:** 10.7759/cureus.115232

**Published:** 2026-08-26

**Authors:** Mauro A Cardenas Rosales, Sergio Miguel Suárez Fuentes, Jazmin Sáenz López, Paola I Cárdenas Rosales, Roberto Guzman Armendariz, Akemy H Tsuyi Loya, Martín Rodríguez-Garza, Cesar Daniel Delgado Calderon

**Affiliations:** 1 Surgery, Hospital General “Presidente Lázaro Cárdenas del Río”, Instituto de Seguridad y Servicios Sociales de los Trabajadores del Estado (ISSSTE), Chihuahua, MEX; 2 General Surgery, Hospital Regional Valentin Gomez Farías, Zapopan, MEX; 3 General Medicine, Autonomous University of Chihuahua, Chihuahua, MEX; 4 School of Medicine, Autonomous University of Chihuahua, Chihuahua, MEX; 5 General Surgery, Instituto de Seguridad y Servicios Sociales de los Trabajadores del Estado (ISSSTE), Monterrey, MEX

**Keywords:** acute pancreatitis, cystogastrostomy, pancreatic necrosis, pancreatic pseudocyst, walled-off pancreatic necrosis

## Abstract

Pancreatic pseudocysts and walled-off pancreatic necrosis are distinct late complications of acute pancreatitis, each requiring different therapeutic approaches. Accurate characterization of pancreatic fluid collections before intervention is essential to minimize treatment failure and procedure-related complications. We present a case of a female patient with a history of severe necrotizing biliary pancreatitis who underwent surgical cystogastrostomy for a presumed pancreatic pseudocyst. During surgery, a retrogastric collection was accessed through the anterior gastric wall, yielding 2,600 mL of fluid with macroscopically apparent debris, followed by cystogastrostomy using an endoscopic linear stapler and laparoscopic cholecystectomy. The initial postoperative course was favorable; however, five days later, the patient developed severe upper gastrointestinal bleeding with a hemoglobin level of 6 g/dL. Urgent endoscopy demonstrated Forrest Ib bleeding at the cystogastrostomy site without successful endoscopic hemostasis, requiring emergency laparotomy, gastrostomy, and surgical hemostasis. The subsequent course was complicated by persistent gastric fistula, tissue lysis, and complete anastomotic dehiscence, requiring multiple surgical interventions, including closure of the posterior gastric defect with an omental patch, diverting jejunostomy, and feeding jejunostomy. After clinical stabilization and enteral nutritional support, delayed intestinal reconstruction was successfully performed. This case highlights the importance of distinguishing pancreatic pseudocysts from walled-off pancreatic necrosis, particularly in patients with a previous episode of extensive pancreatic necrosis. Although delayed imaging may demonstrate a predominantly fluid-filled collection, residual necrotic material may persist and influence treatment outcomes. Current management of walled-off pancreatic necrosis favors a delayed, minimally invasive step-up approach, with endoscopic transmural drainage, followed by necrosectomy when clinically indicated. Careful assessment of the nature and maturity of pancreatic collections before intervention may reduce the risk of severe complications, including bleeding, fistula formation, and anastomotic failure.

## Introduction

Acute pancreatitis is an inflammatory condition of the pancreas that is broadly classified into interstitial edematous and necrotizing pancreatitis according to the presence or absence of pancreatic and/or peripancreatic necrosis [1,2]. Necrotizing pancreatitis may subsequently evolve into organized pancreatic and peripancreatic collections. Among these late complications, walled-off pancreatic necrosis (WON) and pancreatic pseudocyst (PP) represent distinct entities that require different therapeutic considerations [1,2].

WON is defined as an encapsulated collection containing both pancreatic and peripancreatic necrotic tissue, typically developing four weeks after the onset of necrotizing pancreatitis [1,2]. In contrast to a PP, which consists almost exclusively of homogeneous fluid with minimal or no necrotic component, WON contains variable amounts of solid debris and encapsulated organic remnants surrounded by an inflammatory wall composed of granulation and fibrous tissue [1,2]. This clinical, radiological, and pathological distinction is crucial, as the presence of solid material significantly increases the complexity of the collection and may increase the risk of secondary infection.

A considerable proportion of sterile and asymptomatic WON cases may resolve spontaneously with conservative management [2,3]. However, persistent or symptomatic collections may lead to severe complications, including systemic inflammatory response syndrome (SIRS), refractory abdominal pain, nutritional impairment secondary to gastric or biliary outlet obstruction, pancreatic or enteric fistulas, gastrointestinal bleeding, and portal hypertension [2,3].

Historically, open surgical necrosectomy was considered the standard treatment for complicated WON. However, this traditional approach was associated with substantial morbidity and mortality [3,4]. Over the past several decades, the therapeutic paradigm for WON has undergone a substantial shift toward minimally invasive strategies through the step-up approach [3,5-7]. Within this algorithm, endoscopic ultrasound (EUS)-guided drainage has become an important first-line intervention in appropriately selected patients [2,3,5,8].

## Case presentation

A 34-year-old female with no known significant medical history presented to the emergency department with severe, oppressive epigastric pain rated 10/10 on the visual analog scale, radiating to the back in a band-like pattern and accompanied by seven episodes of vomiting containing gastric contents. Following her initial clinical and diagnostic evaluation, she was admitted with a diagnosis of moderate acute pancreatitis according to the Acute Physiology and Chronic Health Evaluation II (APACHE II) [9] and Balthazar classifications (Balthazar grade C) [10], Tokyo grade I acute cholecystitis [11], high-probability choledocholithiasis according to American Society for Gastrointestinal Endoscopy (ASGE) criteria [12], moderate acute cholangitis, probable hydrocholecystitis, and class III obesity according to the World Health Organization (WHO) classification (Table 1) [13].

**Table 1:** Laboratory findings at presentation. Laboratory values obtained at the time of initial presentation, including the corresponding reference ranges and measurement units.

Laboratory test	Patient’s value	Reference range	Units
Hemoglobin	15.9	14 - 18	g/dL
Hematocrit	43	42 - 52	%
Platelets	351	150 - 400	×10³/µL
Leukocytes	17.34	4.5 - 10.5	×10³/µL
Neutrophils	14.9	2.1 - 6.1	×10³/µL
Glucose	181	70 - 110	mg/dL
Blood urea nitrogen	22	5 - 18	mg/dL
Creatinine	0.7	0.7 - 1.2	mg/dL
Amylase	3329	28 - 110	UI/L
Sodium	142	136 - 145	mmol/L
Potassium	4	3.5 - 5.1	mmol/L
Chloride	105	98 - 107	mmol/L
Albumin	4.11	3.4 - 4.8	g/dL
Direct bilirubin	1.8	0.1 - 0.3	mg/dL
Indirect bilirubin	0.42	0 - 0.75	mg/dL
Total bilirubin	2.21	0 - 1	mg/dL
Lactate dehydrogenase	928	135 - 220	UI/L
Alanine aminotransferase	631	0 - 41	UI/L
Aspartate aminotransferase	750	0 - 37	UI/L
Alkaline phosphatase	140	40 - 120	UI/L
Gamma-glutamyl transferase	270	10 - 66	UI/L

**Figure 1:**
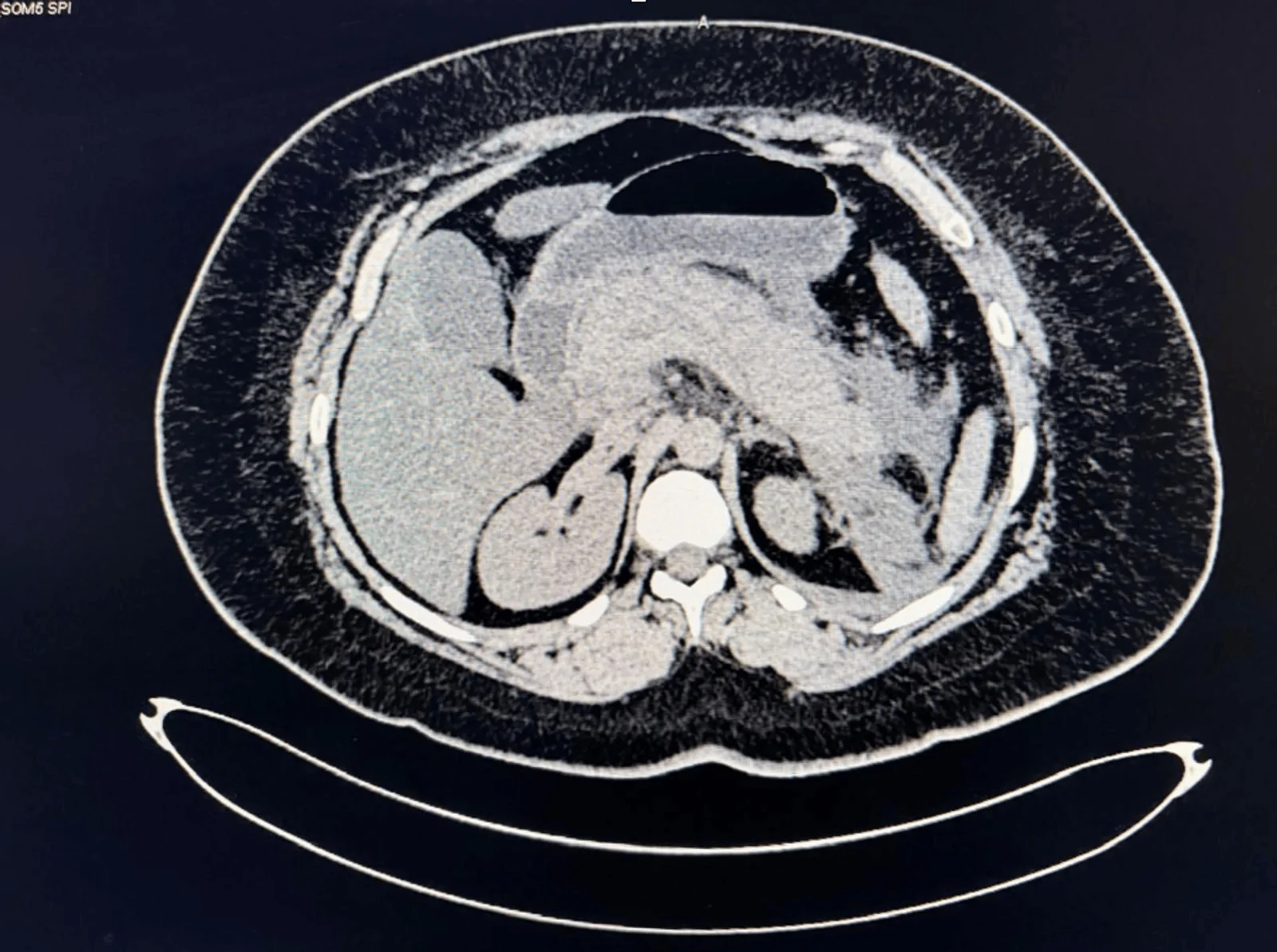
Non-contrast abdominal CT scan performed at presentation. Axial CT image demonstrating significant pancreatic inflammation and peripancreatic fat stranding, consistent with Balthazar grade C pancreatitis.

Upon admission, hepatobiliary ultrasonography revealed grade III hepatic steatosis and a gallbladder with a volume of 87 mL and a thin wall. Two echogenic gallstones measuring 11.3 and 9.5 mm in maximum diameter were identified. As the first choice for radiological diagnosis [6], a non-contrast abdominal computed tomography (CT) scan obtained in the emergency department (Figure 1) and interpreted by a radiologist demonstrated significant pancreatic inflammation and peripancreatic fat stranding, consistent with Balthazar grade C pancreatitis. The common bile duct was dilated to 15 mm, without a visible intraluminal stone. No free intraperitoneal air or free fluid was identified.

**Figure 2:**
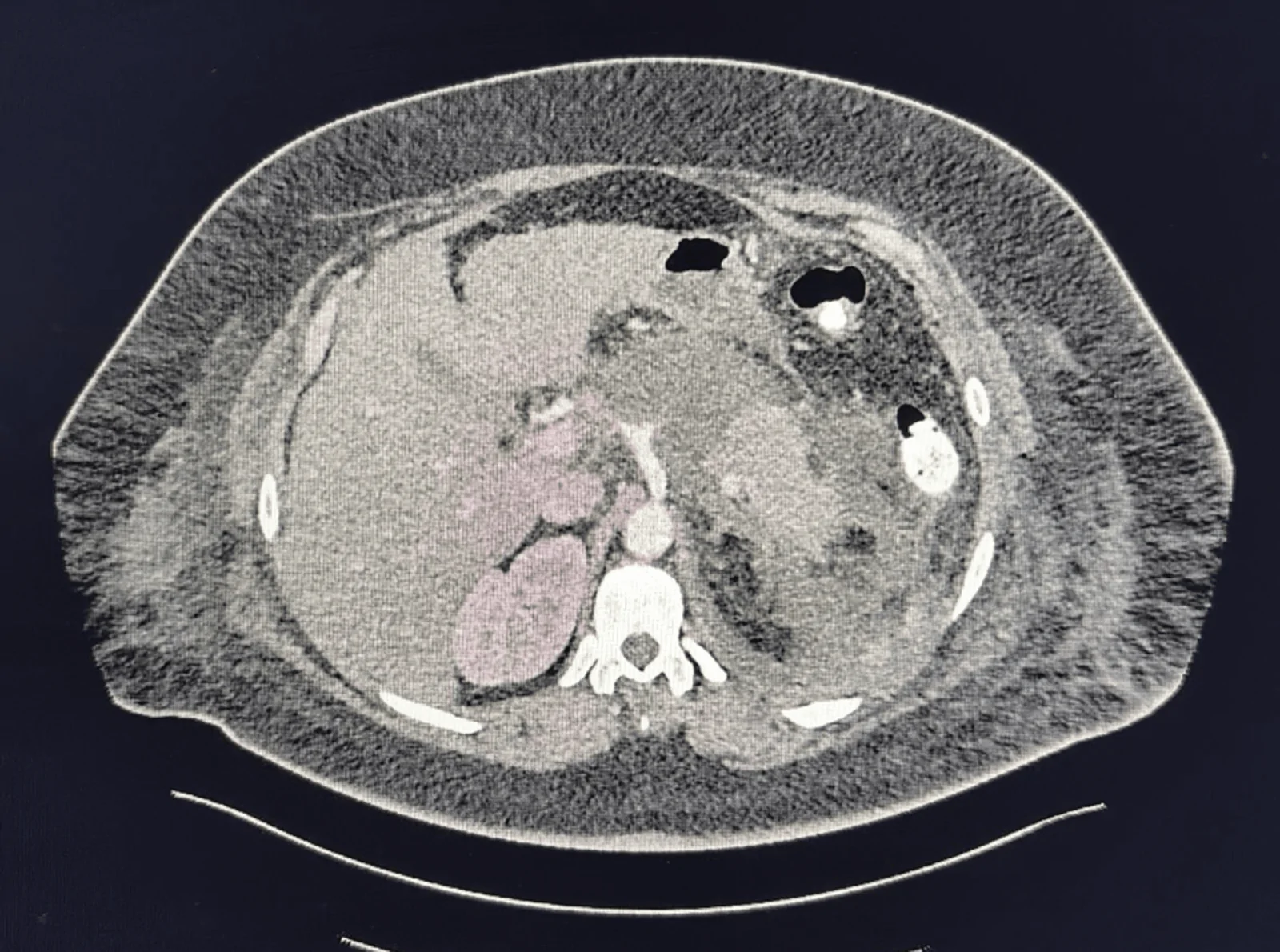
Contrast-enhanced abdominal CT scan, late phase, performed 10 days later after admission. Contrast-enhanced abdominal axial CT scan demonstrating areas of pancreatic necrosis.

A contrast-enhanced abdominal CT scan was performed 10 days after admission (Figure 2), which demonstrated approximately 40% pancreatic necrosis involving the pancreatic body, without evidence of abscess formation or gas within the collection.

During her 10-day hospital stay, the patient received intravenous fluid therapy, which was complicated by the development of bilateral pleural effusions involving less than 10% of the total hemithoracic volume. The effusions were managed conservatively by reducing intravenous fluid administration and initiating diuretic therapy. She was initially kept nil per os and subsequently advanced to a liquid diet, which was well tolerated, followed by progression to a low-fat, non-irritating soft diet. Multimodal analgesia and broad-spectrum antibiotic therapy were administered. Throughout the hospitalization, the patient underwent daily clinical assessments, including evaluation of abdominal pain, vital signs, abdominal findings, and overall clinical status, together with serial laboratory testing to monitor the inflammatory response and organ function. These daily clinical and laboratory evaluations demonstrated progressive improvement in her abdominal pain, inflammatory response, and overall clinical condition. Given her clinical improvement and the absence of evidence of infected pancreatic necrosis or other immediate complications, no intervention was performed during the initial hospitalization. She was discharged with instructions for outpatient clinical and radiological follow-up, including a contrast-enhanced abdominal CT with intravenous and oral contrast approximately six weeks after discharge. However, follow-up was delayed due to administrative and logistical constraints, including limited availability and high demand for outpatient imaging and radiological services. The patient subsequently returned eight weeks after discharge for completion of the requested studies. A dual-contrast CT scan demonstrated a more clearly defined retrogastric collection measuring 15 × 15 × 10 cm, without evidence of displacement or significant compression. A follow-up appointment was scheduled one month later with repeat CT imaging.

At the subsequent follow-up, the patient underwent a second-opinion evaluation by a private physician due to the onset of compressive symptoms, including early satiety and abdominal pain. Based on the clinical presentation and imaging findings, transgastric drainage of the peripancreatic collection was recommended.

**Figure 3:**
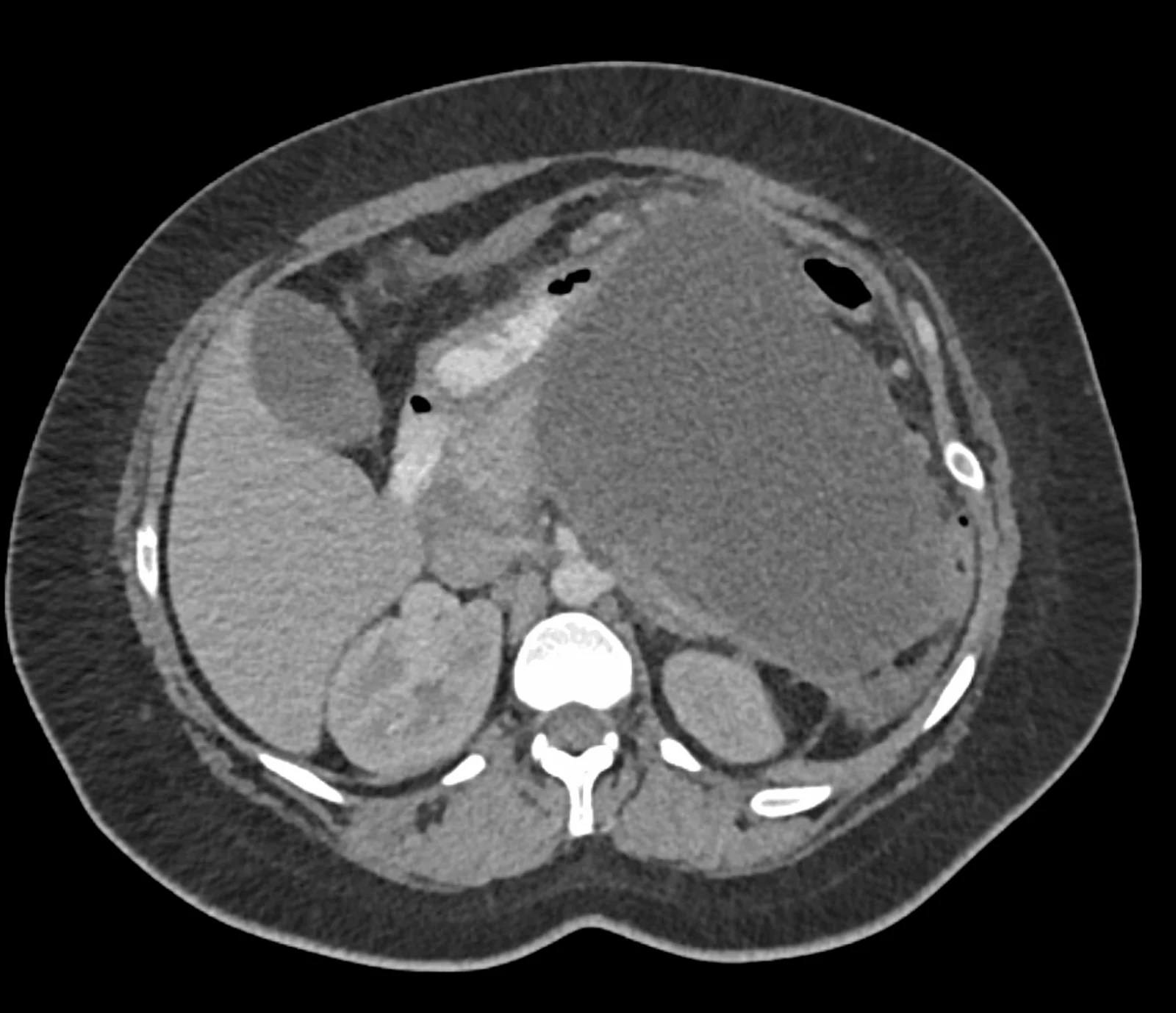
Non-contrast abdominal CT scan obtained four months after discharge. Abdominal CT demonstrating a well-defined encapsulated pancreatic collection.

Four months later, the patient returned for follow-up evaluation. She remained clinically stable, without fever, tachycardia, or other systemic signs suggestive of systemic inflammatory response syndrome (SIRS). As intravenous contrast was not available at the treating institution, a non-contrast abdominal CT scan (Figure 3) was subsequently obtained at a private facility. The CT demonstrated a well-defined, encapsulated peripancreatic collection with a thin wall, accompanied by peripancreatic and pelvic free fluid. Based on its radiological appearance, the collection was initially interpreted as consistent with a PP [14]. Given the persistence of the collection and the patient's compressive symptoms, surgical intervention was planned, consisting of cystogastrostomy and concomitant laparoscopic cholecystectomy.

Surgical treatment

**Figure 4:**
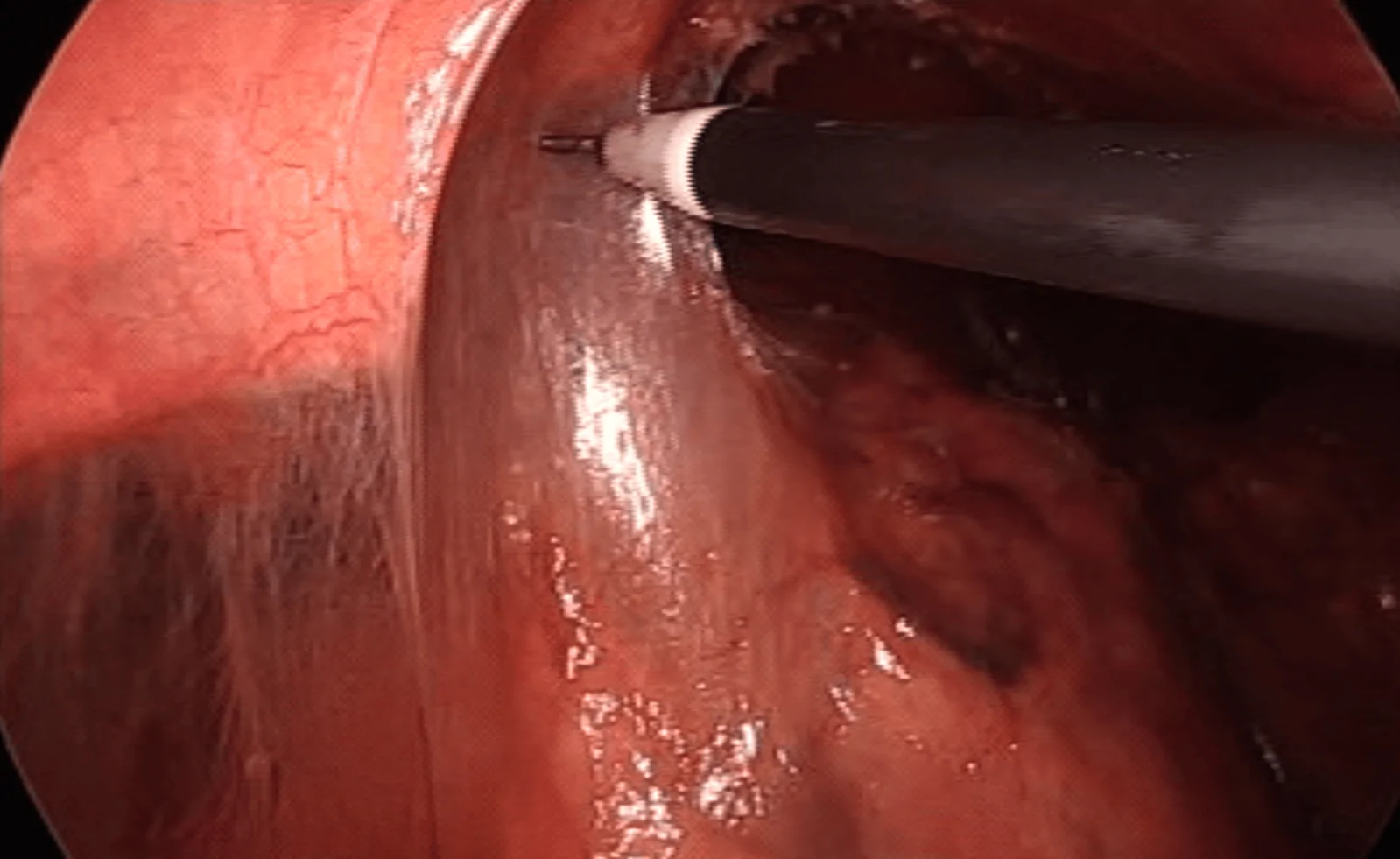
Intraoperative laparoscopic view of the gallbladder. Laparoscopic view demonstrating severe inflammatory changes of the gallbladder, consistent with Parkland grade 5.

**Figure 5:**
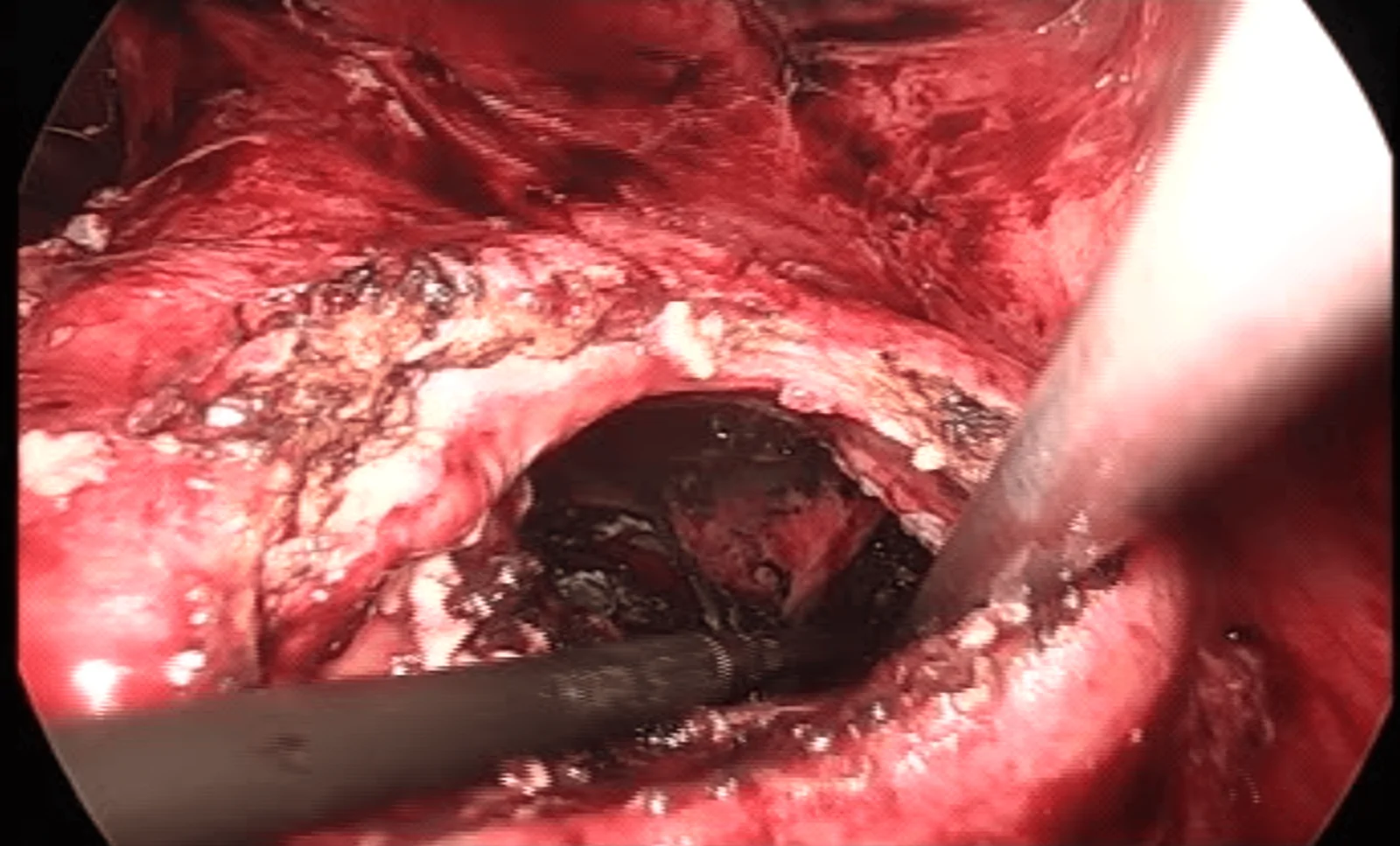
Intraoperative view of the pancreatic collection. Laparoscopic view of the inside of the pancreatic collection, containing necrotic pancreatic and inflammatory material.

**Figure 6:**
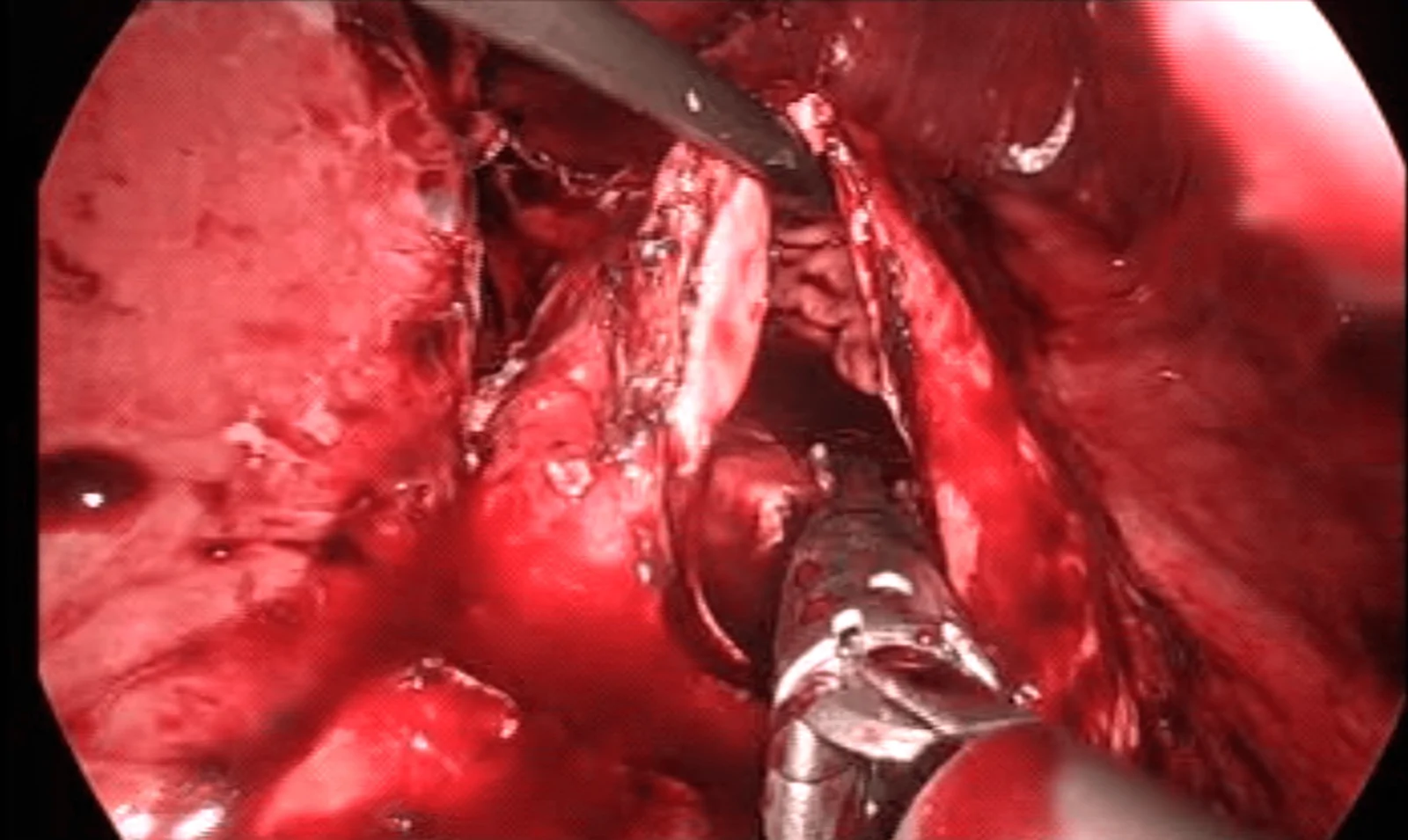
Laparoscopic cystogastrostomy performed using a linear stapler. Intraoperative view demonstrating the creation of a cystogastrostomy using an endoscopic linear stapler for drainage of the pancreatic collection.

The patient underwent surgical cystogastrostomy four months after the last appointment. Intraoperatively, multiple dense adhesions were identified, and the gallbladder was classified as Parkland grade 5 (Figure 4) [15]. Cholecystectomy was performed. Subsequently, through an incision in the anterior gastric wall, the posterior gastric wall was punctured, accessing the retrogastric collection (Figure 5). A total of 2,600 mL of fluid was aspirated, with macroscopically apparent solid debris. A cystogastrostomy was then created using an endoscopic linear stapler (Figure 6).

Clinical course and complications

The patient initially demonstrated an adequate postoperative course during the first five days. However, subsequent laboratory testing revealed a hemoglobin level of 6 g/dL. She developed clinical signs consistent with acute blood loss, including hypotension and tachycardia. No clinical evidence suggestive of infected pancreatic necrosis or SIRS was documented at that time. Urgent upper gastrointestinal endoscopy was performed on the same day and demonstrated massive upper gastrointestinal bleeding, classified as Forrest Ib [16], originating from the anastomotic site. Endoscopic hemostasis was unsuccessful; therefore, the patient was taken emergently to the operating room, where exploratory laparotomy, gastrostomy, and surgical hemostasis were performed. No pancreatic abnormalities were documented in the postoperative surgical report.

One week later, the patient developed persistent clinical and laboratory findings consistent with ongoing blood loss, including anemia with a hemoglobin level of 6.5 g/dL, hypotension, and tachycardia, prompting a second exploratory laparotomy. Intraoperatively, persistent gastric fistula and tissue lysis were identified. The procedure included closure of the posterior gastric wall defect, unroofing of the collection cavity, and surgical hemostasis. No additional pancreatic abnormalities were documented in the operative report.

Two days later, a third exploratory laparotomy was required because of complete anastomotic dehiscence. Primary closure with an omental patch was performed, along with a diverting jejunostomy and a feeding jejunostomy to provide enteral nutrition.

After one month, the patient was discharged in stable condition, tolerating enteral nutrition through the feeding jejunostomy, with a plan for delayed definitive intestinal reconstruction. Two months later, the patient was reassessed in the outpatient clinic and was noted to have improved nutritional and overall clinical status, with adequate tolerance of enteral nutrition and recovery from the previous complications. Based on her clinical improvement and nutritional recovery, definitive intestinal reconstruction was considered appropriate. The patient subsequently underwent exploratory laparotomy through a left lateral approach, followed by mechanical side-to-side jejunojejunostomy reconstruction.

The patient was discharged one week after the surgical procedure, with favorable clinical evolution, tolerating enteral nutrition and without evidence of intestinal leakage.

## Discussion

The revised Atlanta Classification defines a PP as an encapsulated, homogeneous fluid collection with a well-defined inflammatory wall and no significant necrotic component [3]. PPs generally result from disruption of the pancreatic ductal system secondary to pancreatitis, including biliary or alcoholic pancreatitis, trauma, or chronic pancreatitis [1,2,6]. Intervention should generally be avoided during the early phase of acute pancreatitis, as maturation and organization of the fibrous wall require time [1,2]. Premature intervention may increase the risk of treatment failure and other procedure-related complications [2,4].

Indications for drainage

Initial management of pancreatic fluid collections is generally conservative [2,3,5]. Intervention is indicated in the presence of any of the following: persistent symptoms such as refractory abdominal pain, early satiety, or gastric/duodenal outlet obstruction [2,4,6]; local complications such as obstructive jaundice secondary to biliary compression [2,4,6]; clinical or radiological evidence of infection within the collection [3,5,6]; and vascular complications [2,4,6].

The major diagnostic pitfall: Pancreatic pseudocyst vs. walled-off pancreatic necrosis

A critical diagnostic error is to consider PPs and WON as the same entity [1,2]. Although late imaging may demonstrate a predominantly homogeneous, fluid-filled collection that resembles a PP, the underlying history of extensive pancreatic necrosis should raise concern for residual necrotic debris within the collection (Table 2) [1,2].

**Table 2:** Radiological features distinguishing pancreatic pseudocyst from walled-off pancreatic necrosis. Comparison of the radiological findings of pancreatic pseudocyst and walled-off necrosis, including wall characteristics, internal contents, attenuation, and enhancement patterns [14].

Characteristic	Pancreatic pseudocyst	Walled-off pancreatic necrosis
Content	Predominantly homogeneous fluid	Fluid with variable amounts of solid necrotic debris
CT attenuation (HU)	Approximately 0-30 HU, typically homogeneous	Heterogeneous/mixed attenuation, with fluid and higher-attenuation non-liquid components
Wall	Well-defined, usually smooth and relatively thin	Well-defined, usually thicker and potentially irregular
Contrast enhancement	No enhancement of the contents; peripheral wall may enhance	Necrotic contents do not enhance; peripheral wall typically demonstrates enhancement
Solid debris	Absent or minimal	Present in variable amounts
Fat/non-liquid components	Generally absent	May be present, reflecting necrotic pancreatic or peripancreatic tissue
Internal vascularity	Generally absent within the fluid content	Absent within necrotic areas; viable tissue may demonstrate enhancement

Why did simple transmural drainage fail?

In this case, the failure of simple transmural drainage may have been related to the presence of residual necrotic material and the inflammatory environment associated with WON [2,5,7]. Active pancreatic enzymes may contribute to local tissue injury and impaired healing, potentially predisposing to disruption of the gastric staple line and subsequent anastomotic dehiscence, ultimately contributing to the patient's complicated clinical course.

## Conclusions

The primary diagnostic challenge in this case was distinguishing WON from a PP. Substantial initial pancreatic necrosis should raise suspicion for WON, even when delayed imaging demonstrates a predominantly fluid-filled collection. Failure to recognize residual necrotic material may result in inappropriate cystogastrostomy and increase the risk of severe complications, including hemorrhage, impaired tissue healing, and anastomotic dehiscence.

Current management of WON favors a delayed, minimally invasive step-up approach, allowing maturation and liquefaction of the collection before intervention whenever clinically appropriate. EUS-guided transmural drainage is generally preferred as the initial intervention for symptomatic or complicated collections, with subsequent endoscopic or minimally invasive necrosectomy reserved for patients with persistent symptoms, infection, or inadequate clinical response. In this case, EUS-guided drainage was not available at our institution, and referral to an external center was not feasible because of financial limitations; therefore, surgical management was performed. This case emphasizes the importance of accurately distinguishing WON from PP and tailoring treatment to the characteristics of the collection, clinical evolution, available resources, and individual patient circumstances.
